# Domestic Correspondence

**Published:** 1889-05

**Authors:** 


### Domestic Correspondence.

To the Editor:—In view of some late lectures on the germ theory of dental caries, I should like to ask you if the operator who implanted a tooth for Dr. Chas. Andrews, and illustrated his operation in the January *Cosmos*, showing the solid gold bar running across the tops of three adjoining teeth, did not make a fine nest for the cultivation of bacteria? As I see it, many of the forms of bacterial life are without doubt operative in promoting the destruction of teeth. Promoting, not causing, for they are not known to have promoted the destruction of the teeth of early races, nor of savage races of to-day. We do not know that, as a rule, their teeth ever commenced to decay, for the forms of such teeth as we find in the various museums, which forms probably depended upon their density, show that we may say, their structure, as well as their arrangement in the jaws, all tend to their life-long preservation, and they do so tend to-day among the wild and savage races, and among the physically well-developed members of the most civilized races. I do not mean that there are no apparent exceptions to this rule, but, as Darwin puts it, "the fittest individuals of any given tribe survive," and they survive because their members are fittest to do the work required and fittest to repel destructive agencies.

Before a wall of perfect enamel germs are inoperative and powless, so far as we know. Nature's form and arrangement of perfect teeth is such that this wall must be breached before these microscopic enemies can get any foot-hold. In perfect teeth this wall can be broken in two ways, mechanically and chemically. Bacteria certainly do not act chemically; if they act chemically it is a secondary action. Their primary action is probably that of scavengers, but if such multitudes are needed to do the scavenger's work that they fall in their tracks, the products of their living and dying may become what we call the chemical agent that is a solvent, and thus we again approach the question : "which is first, the egg or the hen?" that is, do bacilli produce acids, or acids bacilli. It is well for us to know that these microscopic organisms promote decay, when once decay is started. Is it not therefore better for us to prevent decay from starting?

If all crevices and crannies existing in a tooth can be filled with any indestructible substance; and the surface made flush with the smooth surface of the tooth, so that it may all be kept clean from deposits on every side, that tooth is practically free from decay so long as it is kept clean; but if, on the contrary, a special nest for the breeding, not of pure cultures, but of multifarious bacteria be made, what shall we say? E. A. Bogue.

---

To the Editor :—Thinking that it would be of interest to the profession at large, I desire to place on record in our literature a case of supernumerary teeth, reported at a recent meeting of the Academy of Natural Sciences by Prof. Joseph Leidy, M. D. He exhibited a portion of an adult French skull which had an unusual case of supernumerary teeth. All the teeth were solid and undecayed. It presented an abnormally large central, almost equal in width to a double central. There were also four molars, all well developed and situated in the regular line of the arch. There have been on record many supernumerary molars, but being always outside of the arch, I do not know of any with this perfect arrangement. The skull also presented two other abnormalities, a peculiar pouch-like enlargement of turbinated bones, and a very small jugular foramen on one side, it being nearly obliterated.

Philadelphia, March 5, 1888. L. Ashley Faught.

---

To the Editor :—At the last meeting of the Boston Society of Natural History the president of the society, Prof. F. W. Putnam, in giving an account of an Indian burial place at Winthrop, near

Boston, recently discovered and opened, mentioned one skull that "had a thin copper plate over a portion of the forehead." Cloth, woven from twisted vegetable fiber, covered the plate and the bark of some tree was over that, and fine yellow alluvium mixed with fragments of shells was over all to the depth of two feet to the westward sloping surface.

The vegetable materials in contact and near the plate had been preserved from decomposition; also, the hair, skin and bone beneath the plate as well as the brain, which was dried into a small compact mass, adherent to the bone next under the plate.

The bone was colored green under the plate. Copper salts had, probably, been the preserving power that had warded off the attacks of decomposing bacteria.

I mention this as of general interest to our profession at this time, when copper amalgam seems to have a fresh start, as showing the protective power of copper in one of its salts—the carbonate.

GEO. F. WATERS.

